# Willingness to pay to assess patient preferences for therapy in a Canadian setting

**DOI:** 10.1186/1472-6963-5-43

**Published:** 2005-06-07

**Authors:** Carlo A Marra, Luciana Frighetto, Alan F Goodfellow, Amy O Wai, M Lynn Chase, Ruth E Nicol, Carole A Leong, Sally Tomlinson, Barbara M Ferreira, Peter J Jewesson

**Affiliations:** 1 Pharmaceutical Sciences Clinical Service Unit, Vancouver Hospital and Health Sciences Centre, Vancouver British Columbia, Canada; 2 Faculty of Pharmaceutical Sciences, Uniiversity of British Columbia, Vancouver, British Columbia, Canada

**Keywords:** willingness to pay, outpatient, intravenous, antibiotics

## Abstract

**Background:**

Adult outpatient parenteral antibiotic therapy (OPAT) programs have been reported in the literature for over 20 years, however there are no published reports quantifying preference for treatment location of patients referred to an OPAT program. The purpose of this study was to elicit treatment location preferences and willingness to pay (WTP) from patients referred to an OPAT program.

**Methods:**

A multidisciplinary, single centre, prospective study at a 1000-bed Canadian adult tertiary care teaching hospital. This study involved a WTP questionnaire that was administered over a 9-month study period. Eligible and consenting patients referred to the OPAT program were asked to state their preference for treatment location and WTP for a hypothetical treatment scenario involving intravenous antibiotic therapy. Multiple linear regression analysis was performed to determine predictors of WTP.

**Results:**

Of 131 eligible patients, 91 completed the WTP questionnaire. The majority of participants were males, married, in their sixth decade of life and had a secondary school education or greater. The majority of participants were retired or they were employed with annual household incomes less than $60,000. Osteomyelitis was the most common type of infection for which parenteral therapy was required. Of those 87 patients who indicated a preference, 77 (89%) patients preferred treatment at home, 10 (11%) patients preferred treatment in hospital. Seventy-one (82%) of these patients provided interpretable WTP responses. Of these 71 patients, 64 preferred treatment at home with a median WTP of $490 CDN (mean $949, range $20 to $6250) and 7 preferred treatment in the hospital with a median WTP of $500 CDN (mean $1123, range $10 to $3000). Tests for differences in means and medians revealed no differences between WTP values between the treatment locations. The total WTP for the seven patients who preferred hospital treatment was $7,859 versus $60,712 for the 64 patients who preferred home treatment. Income and treatment location preference were independent predictors of WTP.

**Conclusion:**

This study reveals that treatment at home is preferred by adult inpatients receiving intravenous antibiotic therapy that are referred to our OPAT program. Income and treatment location appear to be independently associated with their willingness to pay.

## Background

Adult outpatient parenteral antibiotic therapy (OPAT) programs have been reported in the literature for over 20 years [1]. These programs have now become an accepted alternative to inpatient therapy for select patients and infection types [2-4]. OPAT programs have been demonstrated to be a safe, effective and acceptable alternative to hospitalization [5-7]. Cost analysis of OPAT programs in the U.S., Canadian and other settings have been reported [5,8-13].

An adult OPAT program was implemented at our hospital in 1995. A cost analysis performed at our institution from 1995 to 1998 showed significant cost avoidance for both the hospital and the Ministry of Health [14]. Informal patient satisfaction surveys have shown that the program has been well received by patients. However, there are no published reports quantifying preference for treatment location of OPAT program patients. Willingness to pay (WTP) is a method of quantifying preference and this methodology is gaining popularity in health care [15-19]. WTP provides a measure of how much an individual values a particular treatment preference.

The objective of this study was to elicit hypothetical infection treatment location preferences, and the willingness to pay for this preferred treatment option from OPAT program referral patients.

## Methods

This was conducted as a multidisciplinary, single centre, prospective study at a 1000-bed Canadian adult tertiary care teaching hospital. Our OPAT program receives about 250 patient referrals and provides approximately 3,000 patient-days of outpatient parenteral therapy per year [20]. General characteristics of patients managed by this service are described elsewhere [14,20]. Adult patients are accepted into the program if they had a proven or suspected infection requiring one or more parenteral antimicrobials for an expected minimum duration of 5 days, are medically stable, have an acceptable venous access, demonstrate a willingness and capability to perform the necessary self-management tasks and live in a suitable home environment with access to a telephone. Once enrolled, the OPAT pharmacist and nurses provide patient teaching, insert the appropriate vascular device, liaise with community nursing personnel, coordinate delivery of drugs and supplies and arranged appropriate patient follow up. These patients are treated in their home environment and return to the hospital periodically for follow-up purposes only. Patients requiring short courses of parenteral antibiotics are typically excluded from the program and are treated as inpatients or managed in a hospital medical daycare setting.

This study was approved by the University Ethics Committee and the Hospital Research Committee.

### Patient enrollment

Patients referred to the OPAT program during the 9-month study (September 1999 – June 2000) were considered eligible for the patient preference assessment. For inclusion into the study, the questionnaire had to be administered to the patient prior to assessment by the OPAT clinical staff for possible inclusion into the program. Only consenting patients were administered the WTP questionnaire. This precaution was taken to avoid the possibility of biased responses from the participants in an attempt to obtain the treatment location of their choice.

### Willingness to pay

Contingent valuation methodology (CVM) was used to quantitatively measure patient preference (i.e. WTP) for intravenous antibiotic treatment location [15-19]. CVM is a survey-based approach for eliciting a consumer's monetary valuations for program benefits for use in cost-benefit analysis. The specific methodology employed was similar to that adopted by Donaldson et al [21]. The consumer utility being measured was compensating variation and the survey measured WTP in the context of program availability. Since the questionnaires were given to individuals undertaking the valuation who were already consumers of the treatment in question (i.e. were receiving parenteral antimicrobials when enrolled) and thus the primary uncertainty at the time of the questionnaire was the probability of treatment course outcomes, an ex-post perspective was adopted [15,16].

The WTP questionnaire consisted of two hypothetical scenarios that were created based upon OPAT program data collected during the period 1995–1998 (see Additional File 1) [14]. Each hypothetical scenario involved an infection that required a 23-day course of intravenous antibiotics (the average duration of therapy for patients enrolled in the OPAT program). The first scenario described a hospital treatment course, while the second scenario described a similar treatment regimen that was administered in the home setting. Using these historic data, the risks associated with each treatment location were also provided to the patient [14]. Patients were asked to specify their preference for treatment location based upon these scenarios. Utilizing open-ended questions, patients were asked to quantify their preference by stating how much they would be willing to pay to obtain treatment in their preferred location.

The survey was initially designed and tested on ten patients, four clinical nurse specialists and two infectious diseases specialists. We utilized comments from these individuals to modify our survey in order to improved readability and understandability.

### Data collection

A single investigator coordinated all patient self-administered WTP questionnaires. Partial completion of the surveys was identified in four of the first eleven patients; thereafter, the investigator examined the questionnaires for completeness at bedside and encouraged patients to provide responses if necessary. If requested by the patient, the questionnaire was read aloud and the investigator recorded the patient's responses. In these cases, the completed questionnaire was subsequently reviewed with the patient to ensure accuracy. Demographic information, socioeconomic data, infection details and WTP for treatment location were collected for all patients.

### Data analysis

Means, medians and ranges for WTP by treatment preference location were determined. Differences in means and medians were testing using t-tests and Mann-Whitney U tests, respectively. To estimate an overall monetary valuation, a total WTP was calculated for patients preferring hospital treatment and a total WTP was also calculated for those preferring home treatment.

A positive association between WTP and income was assessed to determine the construct validity of our questionnaire [22]. WTP was examined for normality using histograms to determine if a natural log transformation was necessary to fit the assumptions of linear regression. Using WTP as the dependent variable, multiple linear regression was utilized. Univariate analyses were performed between each of the possible predictor variables (gender, marital status, level of education, employment, annual household income, infection type) and the dependent variable using ordinary least-squares linear regression.

Variables associated with WTP with a p-value < = 0.10 in the univariate analyses were considered in the multiple linear regression models. Adjusted r^2 ^was calculated for the multivariable models to determine the amount of variance in the outcome variable explained by the predictor variables in the final models. Among significant variables, two-way interactions were investigated. No adjustments were made to p-values to account for multiple comparisons. Studentized residuals and Cook's distance were examined to determine if assumptions of multiple linear regression were violated. Two-sided P values are reported for all analyses. A p value of less than 0.05 was considered to be statistically significant. All analyses were conducted by using SPSS, version 10.

## Results

During the 9-month study period, 131 patients were considered eligible for enrollment in the contingent valuation analysis. Of these patients, 40 were excluded, as the investigator was unavailable to conduct an interview prior to the assessment by the OPAT team, informed consent could not be obtained due to language barriers, decreased cognitive status was evident or patients simply declined to participate. The remaining 91 patients completed the WTP questionnaire.

Patient demographics and socioeconomic status are reported in Table 1. Participants were typically married males in their sixth decade of life with a secondary school education or greater. The majority of participants were retired or were employed with an annual household income of less than $60,000. Osteomyelitis was the most common type of infection for which parenteral therapy was required.

**Table 1:** Patient demographics

**Parameter**	**Value**
**No. of Patients**	91
**Mean age, years **(range)	56 (25–81)
**Gender**	
Male (%)	63 (69)
**Marital Status (%)**	
Married	60 (66)
Divorced	11 (12)
Widowed	9 (10)
Single	10 (12)
**Highest Level of Education (%)**	
Elementary School	7 (8)
Secondary School	29 (32)
Trades/Technical College	34 (36)
University Degree	16 (18)
Post-graduate	5 (6)
**Employment (%)**	
Retired	45 (49)
Employed	29 (32)
Unemployed	16 (18)
Unknown	1 (1)
**Annual Household Income (%)**	
< $20,000	22 (24)
$20,000–39,999	20 (23)
$40,000–59,999	22 (24)
$60,000–79,999	9 (10)
$80,000–99,999	3 (3)
$100,000–149,000	7 (8)
> $150,000	4 (4)
Unknown	4 (4)
**Type of Infection (%)**	
Osteomyelitis	39 (43)
Infected pacemaker/wires	9 (10)
Endocarditis	9 (10)
Wound infection	7 (8)
Abscess	6 (7)
Bacteremia	4 (5)
Other^1^	17 (17)

^1^Meningitis (3), pneumonia (4), infected graft of lower limb (2), line sepsis (2), septic arthritis (2), cellulites (1), pyelonephritis (1), CMV (1), discitis (1)

### Willingness to pay

Of the 91 patients who were enrolled in the study, 87 (96%) indicated a treatment location preference while the remaining four participants had no preference. Of those 87 patients who indicated a preference, 77 (89%) preferred treatment at home while 10 (11%) preferred treatment in hospital. Seventy-one (82%) patients provided an interpretable response regarding WTP for treatment in their preferred location. Of those 16 patients (13 patients with a preference for home therapy vs. 3 patient with a preference for hospital therapy) who did not provide an interpretable response, one registered an astronomically high "protest" WTP far exceeding their ability to pay, while 15 indicated a treatment preference but provided no monetary value.

For those 71 patients who provided an interpretable response, 64 patients preferred treatment at home with a median WTP of $490 CDN (mean $949, range $20 to $6250), and 7 patients preferred treatment in the hospital with a median WTP of $500 CDN (mean $1123, range $10 to $3000). Tests for differences in means and medians revealed no statistically significant differences between WTP values between the treatment locations at the 5% level. The total WTP for the seven patients who preferred hospital treatment was $7,859 versus $60,712 for the 64 patients who preferred home treatment.

**Figure 1:**
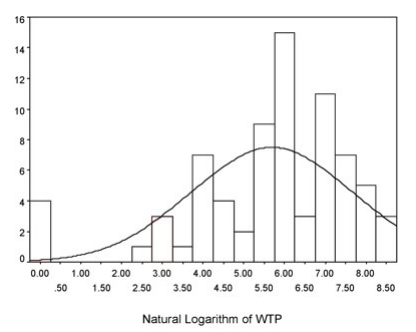


The natural logarithm of WTP values approximated a normal distribution, thus satisfying this assumption of linear regression (Figure 1). Only seventy-five patients (71 patients with an interpretable response plus those 4 patients with no treatment location preference) were included in the regression analysis. Multiple linear regression analysis revealed that income and treatment location preference were independent predictors of WTP (Table 2). There was a trend towards respondents with lower incomes being willing to pay slightly less for their preferred treatment location than those with the highest incomes (p = 0.067). In addition, people who stated preferences were willing to pay significantly more for than those who did not state a preference (p < 0.001). In the multiple linear regression model that included interaction terms (adjusted r2 = 0.543), there was also a significant interaction between income and treatment location preference such that patients with the lowest income were willing to pay significantly more for hospital treatment than for home treatment (p < <0.0001). The fact that there was a significant association between WTP and ability to pay (i.e. higher income) validates the theoretical construct of our survey.

**Table 2:** WTP regression analysis^1,2^

Parameter	β - coefficient	p-value	95% Confidence Interval	
			Lower	Upper
Intercept	0.75	0.35	-0.82	2.32
Income, $ CAN		0.067		
≤ 20,000	-0.991	0.031	-1.89	-0.093
21,000–79,000	-1.011	0.037	-1.96	-0.060
≥ 80,000	Reference			
Location preference		<0.001		
Home	6.10	<0.001	4.58	7.54
Hospital	6.13	<0.001	4.32	7.94
None	Reference			

^1^Dependent variable is the natural logarithm of WTP

^2^Adjusted r2 = 0.478

## Discussion

To our knowledge, this is the first published report quantifying preference for treatment location in an adult OPAT program patients using WTP.

According to our WTP analysis, candidates for the program expressed an overwhelming preference for treatment in the home setting. Our results also demonstrated that the WTP values were similar between those patients who preferred to be treated at home and those who wished to remain in hospital. Accordingly, the total WTP value was greater (in fact, almost 8-fold greater) for those patients preferring treatment at home. This reflects the overall magnitude of societal preference for the management of infectious diseases that require intravenous therapy, but does not require institutionalization.

There were several limitations to this study. We conducted this trial in one adult acute care institution, thus caution must be exercised when attempting to generalize the results to other health care settings involving different patient populations, and other infectious diseases which will require different treatment regimens. We relied on a hypothetical treatment scenario in our attempt to solicit a preference location and willingness to pay for this patient population. As the scenario did not necessarily reflect the treatment that they were about to receive, we must be careful in our extrapolation of the results. Although we acknowledge the potential problems with using such scenarios in CVM, we believe that this effect was minimized by surveying patients who were currently experiencing an infection that initiated a consult from the OPAT team (i.e. the ex-post perspective). We believe that most of these individuals would be able to realistically comprehend the health outcomes described in our scenarios.

We also relied on open-ended technique rather than a bidding-game technique to solicit a WTP value. While the bidding game technique forces an upper and lower limit to the patient response and can be criticized for introducing a starting point bias, the open-ended technique has also been questioned. As described by O'Brien and Viramontes, patient naivety regarding health care costs due to the Canadian universal health insurance environment may lead to an inability to quantify the value of an expected health improvement [16]. The broad range of WTP values provided by our participants may be a reflection of this naivety. In addition, O'Brien and Gafni discuss that open-ended questions often elicit large numbers of non-responses or protest zero responses [21]. Indeed, some patients in our study expressed difficulty in placing a dollar value on their choice of treatment location. In some cases, this appeared to be a protest against the interview question and reflected a concern that their response would be used to determine a future fee for their treatment preference. In other cases, this may have been related to the fact that patients are not typically aware of, nor directly pay for, the costs of health care services in the Canadian health care system. Finally, WTP surveys measure only what a patient claims they are willing to pay for a particular treatment. The magnitude of payment is not necessarily an accurate reflection of what they would actually be willing to pay if they were to encounter the actual scenario.

As expected, ability to pay was associated with WTP and this functioned as a confirmation of construct validity of our questionnaire. Unfortunately, as mentioned by Drummond et al, there is not an actual market for most health programs and, thus, there is no "gold standard" against which one can compare WTP values [22]. It is, therefore, difficult to establish criterion validity in this context.

## Conclusion

This study reveals that the majority, but not all, of adult inpatients receiving parenteral antibiotic therapy who are referred to an outpatient parenteral antibiotic therapy program prefer to be treated at home. Income and treatment location appear to independently predict their willingness to pay.

## Competing interests

This was an unfunded study. The authors declare that they have no competing interests.

## Authors' contributions

CM made substantial contributions to the conception, design, analysis and interpretation of the data; he was involved in the drafting of the article and revising it critically for intellectual content and has given final approval for the current version to be published. LF made substantial contributions to the conception, design, analysis and interpretation of the data; she was involved in the drafting of the article and revising it critically for intellectual content and has given final approval for the current version to be published. AG contributed to the study design, made substantial contributions to data collection, and assisted in the interpretation of the data; he was involved in the drafting of the article, revising it, and has given final approval for the current version to be published. AW contributed to the study conception, design, daily supervision of and participation in data collection, analysis and interpretation of the data; she was involved in the drafting of the article, revising it, and has given final approval for the current version to be published. LC contributed to the study design, data collection and interpretation of the data; she was involved in the drafting of the article and has given final approval for the current version to be published. RN contributed to the study design, data collection and interpretation of the data; she was involved in the drafting of the article and has given final approval for the current version to be published. CL contributed to the study design, data collection and interpretation of the data; she was involved in the drafting of the article and has given final approval for the current version to be published. ST contributed to the study design, data collection and interpretation of the data; she was involved in the drafting the article and has given final approval for the current version to be published. BF contributed to the study design, data collection and interpretation of the data; she was involved in the drafting of the article and has given final approval for the current version to be published. PJ was the coordinating investigator, made substantial contributions to the conception, design, analysis and interpretation of the data; he was involved in the drafting of the article and revising it critically for intellectual content and has given final approval for the current version to be published. All authors take public responsibility for appropriate portions of the content of the manuscript and all authors have read and approved the final manuscript.

## Pre-publication history

The pre-publication history for this paper can be accessed here:



## Supplementary Material

Additional File 1 WTP BMC HSR Version 1 PJJ appendix 1.doc. This file contains the questionnaire used for this study Click here for file

## References

[B1] Stiver HG, Telford GO, Mossey JM, Cote DD, van Middlesworth EJ, Trosky SK, McKay NL, Mossey WL (1978). Intravenous antibiotic therapy at home. Ann Intern Med.

[B2] Kind AC, Williams DN, Persons G, Gibson JA (1979). Intravenous antibiotic therapy at home. Arch Intern Med.

[B3] Poretz DM (1991). Home intravenous antibiotic therapy. Clin Geriatr Med.

[B4] Poretz DM, Eron LJ, Goldenberg RI, Gilbert AF, Rising J, Sparks S, Horn CE (1982). Intravenous antibiotic therapy in an outpatient setting. JAMA.

[B5] Grayson ML, Silvers J, Turnidge J (1995). Home intravenous antibiotic therapy. A safe and effective alternative to inpatient care. Med J Aust.

[B6] Tice AD (1995). Experience with a physician-directed, clinic-based program for outpatient parenteral antibiotic therapy in the USA. Eur J Clin Microbiol Infect Dis.

[B7] Montalto M (1996). Patient's and carers' satisfaction with hospital-in-the-home care. Int J Qual Health Care.

[B8] Balinsky W, Nesbitt S (1989). Cost-effectiveness of outpatient parenteral antibiotics: a review of the literature. Am J Med.

[B9] Williams DN, Bosch D, Boots J, Schneider J (1993). Safety, efficacy, and cost savings in an outpatient intravenous antibiotic program. Clin Ther.

[B10] Chamberlain TM, Lehman ME, Groh MJ, Munroe WP, Reinders TP (1988). Cost analysis of a home intravenous antibiotic program. Am J Hosp Pharm.

[B11] Parker SE, Nathwani D, O'Reilly D, Parkinson S, Davey PG (1998). Evaluation of the impact of non-inpatient i.v. antibiotic treatment for acute infections on the hospital, primary care services and the patient. J Antimicrobiol Chem.

[B12] Thickson ND (1993). Economics of home intravenous services. Pharmacoeconomics.

[B13] Cote D, Oruck J, Thickson N (1989). A review of the Manitoba home i.v. antibiotic program. Can J Hosp Pharm.

[B14] Stiver G, Wai A, Chase L, Frighetto L, Marra C, Jewesson P (2000). Outpatient intravenous antibiotic therapy: The Vancouver Hospital experience. Can J Infect Dis.

[B15] Gafni A (1998). Willingness to pay. What's in a name?. Pharmacoeconomics.

[B16] O'Brien B, Viramontes JL (1994). Willingness to pay: a valid and reliable measure of health state preference. Medical Decision Making.

[B17] O'Brien B, Gafni A (1996). When do the dollars make sense? Toward a conceptual framework for contingent valuation studies in health care. Med Decis Making.

[B18] Bala MV, Mauskopt JA, Wood LL (1999). Willingness to pay as a measure of health benefits. Pharmacoeconomics.

[B19] McIntosh E, Donaldson C, Ryan M (1999). Recent advances in the methods of cost-benefit analysis in healthcare. Pharmacoeconomics.

[B20] Wai AO, Frighetto L, Marra CA, Chan E, Jewesson PJ (2000). Cost analysis of an adult Outpatient Parenteral Antibiotic Therapy (OPAT) Programme. A Canadian teaching hospital and Ministry of Health perspective. Pharmacoecon.

[B21] Donaldson C, Hundley V, Mapp T (1998). Willingness to pay: A method for measuring preferences for maternity care?. Birth.

[B22] Drummond MF, O'Brien B, Stoddart GL, Torrance GW, eds (1997). Methods for the Economic Evaluation of Health Care Programmes.

