# Supplementary material for: Willingness to pay to assess patient preferences for therapy in a Canadian setting
Source: BMC Health Serv Res. 2005 Jun 7;5:43. doi: 10.1186/1472-6963-5-43 (PMC1168895; doi:10.1186/1472-6963-5-43)
Supplement: Additional File 1 — WTP BMC HSR Version 1 PJJ appendix 1.doc. This file contains the questionnaire used for this study [file 1472-6963-5-43-S1.doc]

Additional File 1. WTP Questionnaire

**Patients’ Preferences for Treatment Location for Intravenous Antibiotic Therapy**

You are currently being treated for an infection with an intravenous (IV) antibiotic. There are 2 locations for this treatment which are in the hospital or at home.

In order to determine your preference for treatment location,we would like to describe a hypothetical situation to you. Pretend that your infection would require a further 23 days of IV antibiotic therapy. You would expect to receive the same antibiotic and do equally well in either location. In reality, you and your physician will determine which treatment will be most appropriate for you. We have described the differences between the 2 locations below; all other aspects of care are the same.

**Hospital Care**

You would remain in the hospital for another 23 days of IV antibiotic therapy. A peripherally inserted catheter (PICC) or hickman line would be inserted for IV administration. You would be connected to an IV pole for 30-60 minutes typically 3 times daily and your activities would be limited during these times.

All aspects of care including IV administration of antibiotics would be performed by the hospital staff 24 hours a day. While in hospital, your progress would be followed by your health care team. One in 2 patients would be expected to have a minor catheter problem requiring nursing attention. During your hospital stay, there would be a 1 in 20 chance of developing a second infection from the hospital environment.

There would be no additional charge for a ward hospital bed; however, if you want a semi-private or private room it would cost $50 - $100 per day. Once the antibiotic therapy has been completed, the IV catheter would be removed prior to your discharge from the hospital.

**Home Care**

Once you are clinically stable, you would be discharged home on IV antibiotics for another 23 days. Prior to discharge, a peripherally inserted catheter (PICC) or hickman line would be inserted for IV administration. There is a 1 in 4 chance that you would be connected to a fanny pack size IV pump or a 3 in 4 chance that you would be connected to an IV pole. The pump would be connected to you 24 hours a day and some activities would be limited. The IV pole would be connected to you for 30-60 minutes typically 3 times daily and your activities would be limited during these times.

Initially, you would have community nursing visits at least once daily. You and your family would be asked to learn and take over some aspect of the administration of the IV antibiotic. There would be a 24 hour phone number to call for any problems. One in 2 patients would be expected to have a minor catheter problem requiring nursing attention. There would be a 1 in 5 chance of returning to the hospital clinic or the emergency department for any problems and a 1 in 20 chance of having to be readmitted to the hospital due to complications of the IV antibiotic therapy.

You would come to the hospital clinic once weekly to be monitored by the health care team. Once the antibiotic therapy has been completed, the IV catheter would be removed by the IV nurse during your clinic visit.

**Your answers to the following questions will remain anonymous and will not be provided to the Home IV Antibiotic Program Team prior to the completion of your IV antibiotic therapy. Your responses will not in any way affect your eligibility for enrolment into the Home IV Antibiotic Program.**

**1**. Which location for IV antibiotic therapy do you prefer? (please check the appropriate box)

❑ Hospital - if you prefer hospital care, **go to question 2**

❑ Home - if you prefer home care, **go to question 3**

❑ No preference - if you have no preference, **go to question 5**

**2**. One way of measuring the value you place on your choice of hospital care rather than home care is to ask you how much you would be willing to pay in order to obtain your choice.  **Of course, both types of care are free and will stay free. We believe that people should not have to pay for health care.** This is just a way of measuring how strongly you feel about having hospital care rather than home care and we would never ask you to pay such amounts. So, please **imagine** that you have to pay. There are no right or wrong answers. The amount you choose could be large or small. We are interested in your views.

What is the maximum amount of money you would be prepared to pay to receive hospital care rather than home care for 23 days of IV antibiotic therapy? *(please write your answer in the space below. One way to think of this is to imagine you are at an auction at which the most you would pay for an item shows the importance you place on that item. How far are you prepared to go?)*

$__________

NOW GO TO QUESTION 4

**3**. One way of measuring the value you place on your choice of home care rather than hospital care is to ask you how much you would be willing to pay in order to obtain your choice.  **Of course, both types of care are free and will stay free. We believe that people should not have to pay for health care.** This is just a way of measuring how strongly you feel about having home care rather than hospital care and we would never ask you to pay such amounts.

So, please **imagine** that you have to pay. There are no right or wrong answers. The amount you choose could be large or small. We are interested in your views.

What is the maximum amount of money you would be prepared to pay to receive home care rather than hospital care for 23 days of IV antibiotic therapy? *(please write your answer in the space below. One way to think of this is to imagine you are at an auction at which the most you would pay for an item shows the importance you place on that item. How far are you prepared to go?)*

$__________

**4**. Please state below why you are willing to pay such an amount for the care you chose?

**5**. To help us better understand your opinions, please answer the following:

Age:_____ Gender: ❑ male ❑ female

Marital Status: ❑ single ❑ married ❑ divorced ❑ widowed

Highest Level of Education: ❑ elementary school

❑ secondary school

❑ trades/technical college

❑ university degree

❑ post-graduate degree

Occupation: ❑ retired

❑ unemployed

❑ employed - please specify ________________________

Total Annual Household Income: ❑ less than $20,000

❑ $20,000 - $39,999

❑ $40,000 - $59,999

❑ $60,000 - $79,999

❑ $80,000 - $99,999

❑ $100,000 - $149,999

❑ more than $150,000

Please specify your type of infection: ____________________________

Have you previously been enrolled in a Home IV program: ❑ Yes ❑ No

Do not write your name on this questionnaire. Your responses will remain anonymous.

Thank you for your time.
